# How Language Attitudes Shape French Language Achievement: The Mediating Role of Student Engagement

**DOI:** 10.3390/bs16071255

**Published:** 2026-07-22

**Authors:** Jiarui Gong, Xinli Chen, Yinuo Li

**Affiliations:** 1 School of Humanities, Tsinghua University, Beijing 100084, China; gongjr25@mails.tsinghua.edu.cn; 2 School of Foreign Languages, Huazhong University of Science and Technology, Wuhan 430074, China; rosalynde@hust.edu.cn

**Keywords:** language attitudes, student engagement, French achievement, positive psychology, Control-Value Theory

## Abstract

Drawing on positive psychology, this study investigated the relationships among language attitudes, student engagement (including its cognitive, emotional, behavioral, and social dimensions), and academic achievement in French. A total of 335 Chinese junior high school learners of French as a foreign language (FFL) completed a French proficiency test and filled out validated scales measuring language attitudes and student engagement. Correlation analyses revealed significant positive associations across all three constructs, with effect sizes ranging from small to large. Structural equation modeling (SEM) further demonstrated that: (1) language attitudes were significantly and positively associated with all four dimensions of student engagement; (2) of the four dimensions, only behavioral engagement showed a significant positive path to French achievement and emerged as the only significant positive mediator of the attitudes–achievement relation, with the direct path from language attitudes to achievement being non-significant; (3) the indirect effects through cognitive, emotional, and social engagement were non-significant. These findings contribute to the further examination and extension of Control-Value Theory (CVT) and offer practical implications for FFL pedagogy.

## 1. Introduction

Driven by the socio-psychological and positive psychology frameworks in Second Language Acquisition (SLA), recent scholarship has foregrounded individual learner differences. Within this shift, language attitudes have emerged as a pivotal variable (Li & Li, 2024), constituting a complex socio-psychological construct that captures core cognitive and evaluative orientations toward different languages and their speakers (Myers-Scotton, 2006; Adler & Plewnia, 2018). Studies indicate that positive attitudes not only guide learners’ initial language choices (Hudgens Henderson et al., 2020) but are also closely associated with their subsequent learning behaviors and scholastic outcomes (Gardner, 1988; Smith, 1971; Botes et al., 2020; Masgoret & Gardner, 2003).

Of the numerous learning behaviors shaped by language attitudes, student engagement serves as a critical observable proxy for gauging behavioral performance and forecasting academic achievement (S. Hu & McCormick, 2012; Reeve & Lee, 2014). Given its malleability (Y. Guo et al., 2024; Q. Zhang & Wang, 2023; J. Xu et al., 2023) and its close association with learners’ psychological characteristics and ultimate attainment (H. Xu, 2023), student engagement represents a key behavioral pathway linking language attitudes to foreign language (FL) achievement. While prior empirical studies have separately confirmed that both language attitudes and student engagement can significantly predict second language (L2) achievement (Li & Wei, 2025; Zhu et al., 2022), research focusing on how the two interact to affect learning outcomes remains limited. In particular, few scholars have systematically examined whether language attitudes exert an indirect effect on FL achievement through student engagement—a proximal learning variable—within a unified theoretical framework.

Furthermore, student engagement is not a unitary construct; rather, it is composed of cognitive, emotional, behavioral, and social dimensions (Hiver et al., 2021). The mechanisms through which these dimensions influence academic outcomes may differ substantially. However, most studies have either treated student engagement as a global variable (e.g., Li & Feng, 2025; Lü & Yang, 2013) or focused solely on one or two specific dimensions (e.g., J. Guo et al., 2021; J. Guo et al., 2022). Such approaches may obscure the heterogeneity of its internal structure, masking differences across dimensions and limiting the precision of the findings. Moreover, related research has predominantly focused on university students or centered on English-as-a-foreign-language (EFL) contexts, while comparatively neglecting learners of languages other than English (LOTE) at the basic education level (Shu, 2021; J. Xu, 2021).

Therefore, drawing upon the Control-Value Theory (CVT) as the theoretical framework, and based on data from 335 Chinese junior high school students, this study examines whether and how the four dimensions of student engagement each mediate the relationship between language attitudes and French achievement. In doing so, it aims to make three interconnected contributions to the existing literature: (1) theoretically, it extends the application of CVT to the context of French as a foreign language (FFL) by linking subjective language attitudes, multidimensional engagement, and academic achievement; (2) methodologically, it unpacks the “black box” of student engagement by testing the distinct mediating effects of its four dimensions (cognitive, emotional, behavioral, and social), rather than treating it as a single global construct; and (3) practically, it provides targeted, dimension-specific intervention strategies for the LOTE pedagogy in basic education.

## 2. Literature Review

### 2.1. Control-Value Theory

Rooted in educational psychology (Pekrun, 2006), CVT has gained substantial traction in the study of L2 learning emotions (Li, 2020; Li & Dewaele, 2020). This theory posits that individuals’ control appraisals (judgments of personal competence) and value appraisals (judgments of the task’s significance) serve as the proximal antecedents of academic emotions (e.g., enjoyment, anxiety), which then influence academic achievement by affecting learners’ cognition (e.g., self-regulation), motivation, and behavior (e.g., learning strategies, student engagement) (Pekrun & Stephens, 2010). Building on this, Li and Li (2024) integrated language attitudes into CVT as value appraisals specific to language learning, confirming that language attitudes influence FL achievement through the mediation of emotions. Language attitudes refer to individuals’ overall value evaluations and behavioral tendencies toward a particular language, which are generally considered to comprise three dimensions: cognitive, affective, and behavioral (Garrett, 2010). The cognitive dimension refers to individuals’ perceptions, understandings, beliefs, and evaluations of a language, involving assessments of its intrinsic and extrinsic values. The affective dimension captures emotional responses toward the language (e.g., like vs. dislike, intimacy vs. distance). The behavioral dimension represents tendencies to act in specific ways regarding the language (Garrett, 2010).

**Figure 1:**
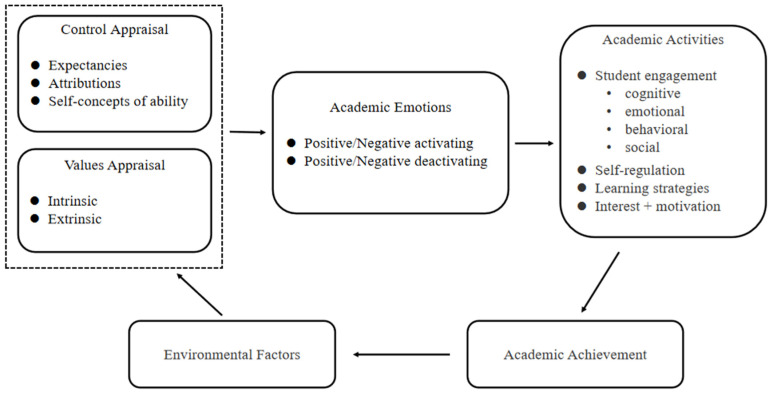
The CVT framework. Source. Adapted from Pekrun (2006).

Huguet (2006) argued that “language learning will rarely occur if subjects do not show positive attitudes to the language in question and the lessons where it is taught” (p. 414). In other words, students’ behaviors align with their attitudinal evaluations of the language and the language course. This indicates that language attitudes manifest as learning behaviors (Li & Wei, 2022). Meanwhile, as a crucial variable explaining differences in learning behaviors, student engagement has garnered widespread academic attention in recent years, influenced jointly by positive psychology and educational psychology. An increasing number of studies have found that student engagement both reflects learners’ attitudinal and behavioral investment and predicts academic achievement (Kuh et al., 2008; Sulis & Philp, 2021; Yuan & Lo Bianco, 2022). Student engagement comprises behavioral engagement (including task participation and sustained effort), cognitive engagement (the level of metacognitive strategy use and deep processing capabilities), emotional engagement (involving emotional experiences and feedback mechanisms during learning), and social engagement (manifested as autonomous regulation in pedagogical interactions) (Hiver et al., 2021; Li & Li, 2024; Philp & Duchesne, 2016; Reeve & Tseng, 2011). It is also a prerequisite for effective learning (Oga-Baldwin, 2019) and a primary component of learner well-being (Li & Li, 2024; Seligman, 2018). Within the CVT framework (Figure 1), academic activities—most notably student engagement—form the essential pathway through which academic emotions impact learning achievement. A closer look at this dynamic reveals that favorable language attitudes manifest when learners positively appraise a language’s intrinsic (e.g., enjoyment) and extrinsic (e.g., functionality) value. Such appraisals elicit corresponding positive emotions that empower students, fueling their multifaceted engagement, culminating in enhanced FL attainment. (Li & Li, 2024). Consequently, engagement operates as a critical mediator connecting psychological states to actual learning gains.

### 2.2. Language Attitudes and FL Achievement

Aligning with the aforementioned definitions, current research on the relationship between language attitudes and FL achievement primarily follows two directions. The first explores the direct correlation between these variables. Multiple empirical studies indicate that students with positive language attitudes tend to achieve better outcomes in language learning. For example, Makrami (2011) found that Saudi university students learning English for specific purposes scored higher in tests when exhibiting favorable dispositions. Similarly, Clément et al. (1994) identified a positive correlation between Hungarian EFL learners’ attitudes toward English and their English academic achievement. Malallah (2000) also showed that higher English test scores generate a greater need to learn English, which in turn fosters more positive attitudes toward English learning among students. Furthermore, in language learning, student achievement can be measured by assessing their proficiency in the target language. For example, Scherer and Wertheimer (1964) found that American university students held positive attitudes toward German and their own German-speaking skills, which highly correlated with their overall German proficiency.

The second direction investigates the indirect relationship between language attitudes and achievement, namely, the influence of other potential underlying factors. Smith (1971) pointed out that language attitudes are related to motivation (instrumental or integrative) and emotions (positive or negative), both of which have proven to be significant determinants of language achievement (Botes et al., 2020; Li & Wei, 2022; Masgoret & Gardner, 2003). Building on this, Li and Li (2024) discovered empirically that emotions play a mediating role between language attitudes and FL achievement.

Despite these findings on the relationship between language attitudes and L2 achievement, current research still presents certain limitations. First, the field of SLA seems to show a pronounced bias in favor of English. Empirical research targeting Romance languages, particularly French, remains relatively scarce, which to some extent weakens the cross-linguistic explanatory power of current theoretical models. Although a few studies have demonstrated that positive attitudes similarly promote academic achievement in LOTE such as Spanish, German, and French (Hsieh, 2008; Flores et al., 2025), empirical data on these non-English contexts are still limited.

Second, regarding the potential factors influencing the attitude-achievement relationship, the limited number of existing studies have a narrow scope. They mostly concentrate on the impact of academic emotions, neglecting the role of student engagement in this process. However, student engagement is actually a critical mediating variable between language attitudes and academic achievement. As indicated by CVT, the effect of academic emotions on learning achievement must be realized through specific learning behaviors. Because student engagement is a more direct behavioral manifestation, it exerts a more proximal impact on learning outcomes. Therefore, incorporating student engagement into the research scope enables a more comprehensive explanation of the core question of how language attitudes affect learning achievement.

### 2.3. Language Attitudes and Student Engagement

Regarding the association between language attitudes and student engagement, direct research remains scarce. Rather than centering specifically on student engagement, some scholars have explored the broader concept of learning behavior. They posit that, within the field of SLA, language attitudes can influence psychological processes—such as emotions and motivation—which in turn shape language learning behaviors (Gardner, 1988; Smith, 1971). Li and Wei (2025) elaborated on this transmission mechanism, noting that learning behaviors align with students’ attitudinal evaluations of the target language and its corresponding courses. Ultimately, these attitudes can translate into specific learning behaviors through cognitive, affective, and evaluative processes. For example, a student who finds a course interesting (cognition) may experience a sense of enjoyment (emotion), come to view the learning experience as worthwhile (evaluation), and eventually engage more actively in classroom activities (behavior).

Altogether, these insights offer promising theoretical backing to the pathway linking language attitudes to student engagement. Conceptually, this alignment appears coherent: engagement encapsulates the interest, active participation, and cognitive resources learners invest in tasks (Newman et al., 1992). Such characteristics resonate with the aforementioned process. Several scholars further conceptualize engagement as a malleable state, continuously negotiated through various structural and psychological variables (Y. Guo et al., 2024; Q. Zhang & Wang, 2023; J. Xu et al., 2023). Language attitudes, encompassing cognitive, behavioral, and affective dimensions, represent exactly the type of internal psychological state capable of driving this malleability. Moreover, given their striking structural alignment with the sub-dimensions of engagement, these attitudinal components may influence both the initiation and persistence of such behaviors. Therefore, it is theoretically plausible to suggest that a meaningful correlation could be observed between language attitudes and student engagement. Nevertheless, further empirical research is warranted to thoroughly understand and validate this association.

Furthermore, existing research within the CVT framework is predominantly based on adult college students (J. Xu et al., 2023; Li & Han, 2022; Wei et al., 2025), whereas the present study focuses on early adolescents. Compared to adults, early adolescents may lack sophisticated capacities for self-reflection and motivational regulation. Consequently, their language attitudes are likely to influence student engagement more directly. For example, lower secondary school students who find French interesting (the affective dimension of language attitudes) may immediately exhibit higher classroom participation (the behavioral dimension of student engagement) without engaging in complex motivational regulation. This direct mechanism of action is likely to be more pronounced among early adolescents.

### 2.4. Student Engagement and FL Achievement

Engagement has been proven to be closely related to a variety of positive learning outcomes (Fredricks et al., 2016; Schlenker et al., 2013; M.-T. Wang & Fredricks, 2014; M.-T. Wang & Holcombe, 2010). While engagement possesses certain domain-general characteristics, such as psychophysiological arousal, attention, and metacognitive awareness, domain-specific engagement dimensions can provide deeper insights into particular academic subjects (Sinatra et al., 2015). Based on this premise, numerous studies have been dedicated to exploring engagement in the fields of science, technology, engineering, and mathematics. In contrast, research in the field of SLA has received relatively less attention (Zhu et al., 2022; Mercer, 2019; Oga-Baldwin & Fryer, 2018; Philp & Duchesne, 2016). Given the critical role of student engagement in academic success, an in-depth exploration of this concept within the SLA domain will help better elucidate its specific functions in this field.

Furthermore, although research on the relationship between student engagement and achievement within the Chinese context has gradually gained traction, existing studies predominantly focus on college students and their EFL learning. In contrast, research on LOTE learners at the basic education level remains scarce. For example, J. Guo et al. (2021) conducted an in-depth investigation into emotional engagement in college students’ EFL learning, revealing its relationship with academic achievement. The study found that internal emotional engagement (e.g., learning interest, self-confidence, and subjective value endorsement) was significantly and positively correlated with English test scores, whereas external emotional engagement (e.g., school satisfaction, sense of belonging, and teacher identification) exerted its influence indirectly through the mediating effect of internal emotion. Similarly, within multi-interaction environments, J. Guo et al. (2022) demonstrated that behavioral engagement is a significant positive predictor of English achievement. Although these studies enrich the empirical foundation for college students’ EFL engagement, they also limit the comprehensive understanding of the motivational mechanisms driving learners across different age groups and linguistic backgrounds. To some extent, this limitation constrains the optimization of multilingual pedagogical practices within the basic education sector.

## 3. Rationale and Research Questions

Despite the progress in existing literature, several critical gaps remain. The empirical foundation regarding the relationship between language attitudes and student engagement remains underdeveloped, with few studies comprehensively examining the relational mechanisms among these three variables (language attitudes, student engagement, and French achievement). Elucidating these mechanisms is crucial for understanding how FL academic achievement is shaped, carrying profound theoretical and practical significance. Furthermore, current research notably overlooks early adolescent FL learners. Given that this demographic is in a critical period for language learning (Lou et al., 2024), and their cognitive, evaluative, and affective responses to languages are still in their formative stages, investigating this group holds unique exploratory value. Finally, a pronounced linguistic bias persists within the current SLA field. The inadequate focus on French constrains the cross-contextual applicability of existing findings. Conversely, an in-depth investigation into how FFL acquisition mechanisms impact academic achievement not only assists in optimizing pedagogical intervention strategies for the FFL learners worldwide, but also contributes to the promotion of multilingual education at the basic education level and fosters broader cross-cultural communication within the Francophone world (La Francophonie).

Drawing upon the aforementioned theoretical framework and empirical literature, the present study proposes the following research questions:

(1) What are the interrelationships among language attitudes, student engagement, and French achievement?
(2) Do the various dimensions of student engagement mediate the relationship between language attitudes and French achievement?

## 4. Methodology

### 4.1. Participants and Context

The participants in this study were junior high school students (Grades 7 to 9) from six middle schools across four cities in China (Wuhan, Chongqing, Chengdu, and Nanchang). All participants were FFL learners. Despite differences in their educational levels, all participants used the same textbooks and followed similar curricula. The study was officially conducted following institutional approval from the participating schools, informed consent from the guardians, and informed assent from the students themselves. This process yielded a final valid sample of 335 participants for analysis. The mean age of the participants was 13.03 years (SD = 0.78). The sample comprised 137 males (40.90%) and 192 females (57.31%), with 6 participants (1.79%) choosing not to disclose their gender.

### 4.2. Instruments

#### 4.2.1. Language Attitudes Scale

The *Language Attitudes Scale* was adapted from the instrument developed by Li and Wei (2025), which is based on the three-dimensional theoretical structure of language attitudes. It was modified to suit FFL learners (see Appendix A). Specifically, references to various languages in the original scale (i.e., [your ethnic language/dialect/Putonghua/English]) were uniformly replaced with “French” across all items. Prior to formal data collection, all items were reviewed by an expert panel (two applied linguists and one educational psychologist) to evaluate the content validity and cultural appropriateness of each item for the FFL context. Subsequently, a pilot study was conducted with 50 FFL students to check item clarity and response patterns. These students were not part of the final sample. Minor wording adjustments were made based on the pilot feedback. The adapted scale utilizes a 7-point Likert scale and comprises 10 items. Of these, 3 items measure the cognitive dimension of language attitudes, 3 items measure the affective dimension, and 4 items measure the behavioral dimension. In the present study, this scale demonstrated high reliability, with an internal consistency (Cronbach’s α) of 0.934. The construct validity was adequate, as suggested by the fit indices: CFI = 0.946 > 0.90, TLI = 0.924 > 0.90, SRMR = 0.060 < 0.08, and RMSEA = 0.086, which falls below the more lenient cutoff of 0.10 originally suggested by L. Hu and Bentler (1995) but exceeds the stricter 0.06 threshold recommended in their later, more widely cited work (L. Hu & Bentler, 1999). Notably, the RMSEA value of 0.086 serves as a marginal fit index, sitting at the upper limit of acceptable fit. This slightly elevated value is likely attributable to the relatively small number of items (10 items) and the modest sample size (N = 335). Kenny et al. (2015) indicate that RMSEA tends to be upwardly biased in models with limited sample sizes, recommending that model fit should be evaluated comprehensively with theoretical justification and multiple fit indices. Importantly, the remaining fit indices (CFI = 0.946, TLI = 0.924, SRMR = 0.060) all met or exceeded conventional thresholds, collectively supporting the adequacy of the measurement model. Therefore, the scale was considered adequate for subsequent analyses.

#### 4.2.2. Student Engagement Scale

The *Student Engagement Scale* was adapted from the *Foreign Language Engagement Scale* developed by Li et al. (2023) to suit FFL learners (see Appendix B). Specifically, the word “English” in the original items was changed to “French”. Following this modification, the scale underwent the exact same adaptation procedure described in Section 4.2.1 (i.e., expert panel review and pilot testing) to assess content validity and clarity. All items requiring reverse scoring were appropriately reverse-coded prior to data analysis. The adapted scale utilizes a 5-point Likert scale and contains 15 items measuring four dimensions: cognitive engagement (4 items), emotional engagement (3 items), behavioral engagement (5 items), and social engagement (3 items). This scale exhibited high reliability in this study, with an internal consistency (Cronbach’s α) of 0.938. The construct validity was satisfactory, with the following fit indices: CFI = 0.961 > 0.90, TLI = 0.951 > 0.90, SRMR = 0.042 < 0.08, and RMSEA = 0.059 < 0.10.

#### 4.2.3. French Achievement

This study employed a standardized final French academic achievement test to measure students’ learning outcomes. The test papers were uniformly developed by the French teaching and research groups across all participating schools, in accordance with the junior high school requirements stipulated in the *National French Curriculum Standards*. The jointly designed paper was scored out of 100 points and primarily assessed students’ comprehensive French language proficiency. It consisted of core sections including listening (20%), vocabulary and grammar (40%), and reading and writing (40%). The item difficulty was strictly adapted to the syllabus of each grade level. Prior to administration, the test content was reviewed by three senior French teachers to support the content relevance and instructional appropriateness of the examinations.

The three grade-specific tests demonstrated reliable internal consistency, with Cronbach’s α of 0.854 for Grade 7, 0.883 for Grade 8, and 0.870 for Grade 9. To reduce the potential influence of grade-specific test difficulty and syllabus coverage, raw French achievement scores were standardized within each grade level (i.e., converted to z-scores within each grade group) before being entered into the subsequent analyses. The examinations were uniformly organized and administered by each school, with grading conducted by teachers who had received standardized training on the scoring rubrics to promote consistent application of the grading criteria.

### 4.3. Data Analysis

Descriptive statistics, reliability analyses, and normality tests were conducted using SPSS 31. Subsequently, confirmatory factor analysis (CFA) for each measurement instrument was performed using Mplus 8.3. To address Research Question 1, Pearson correlation analyses were conducted on all variables using SPSS 31. According to Cohen et al. (2018), correlation coefficients ranging from 0.10 to 0.30 were interpreted as small effects, those from 0.30 to 0.50 as moderate effects, and those greater than 0.50 as large effects. To address Research Question 2, path analysis was conducted using Mplus 8.3 to examine the mediation model involving language attitudes, student engagement, and French achievement. The model fit for both the CFA and the mediation models was evaluated based on the following indices: χ^2^/df < 3, CFI > 0.90, TLI > 0.90, SRMR < 0.08, and RMSEA < 0.10 (L. Hu & Bentler, 1995). Regarding missing data, the multiple imputation (MI) method was employed for the analyses conducted in SPSS, while the full information maximum likelihood (FIML) estimation method was utilized to handle missing values in the Mplus analyses.

## 5. Results

### 5.1. Descriptive Statistics

Table 1 summarizes the descriptive statistics and normality test results for all variables. The data for all variables exhibited a normal distribution (–3 < skewness < 3; –3 < kurtosis < 3; Hair et al., 2019). French achievement is reported as raw scores in Table 1 for interpretability, whereas all subsequent analyses used grade-standardized z-scores. Based on the mean scores, participants held relatively positive attitudes toward French. Furthermore, their student engagement (including all sub-dimensions) and French proficiency levels were above average.

### 5.2. Correlation Analysis

As shown in Table 2, the Pearson correlation analyses revealed significant correlations among all variables (*p* < 0.001). Specifically, the correlations between French test scores and the other variables (language attitudes, total student engagement, and its sub-dimensions) ranged from small to moderate effects (r = 0.248 to 0.382). Meanwhile, the correlations between language attitudes and the remaining variables ranged from moderate to large effects (r = 0.487 to 0.769).

To further evaluate the precision of these associations, 95% confidence intervals (computed via Fisher’s r-to-z transformation) for the correlations central to the study’s research questions are reported in Table 3. Among the four engagement dimensions, their correlations with French achievement were comparable in magnitude (cognitive r = 0.366, 95% CI [0.268, 0.457]; emotional r = 0.296, 95% CI [0.194, 0.391]; behavioral r = 0.364, 95% CI [0.266, 0.455]; social r = 0.248, 95% CI [0.144, 0.346]), with language attitudes correlating with French achievement at r = 0.328, 95% CI [0.228, 0.422]. Across all key correlations, the confidence intervals were relatively narrow, indicating that these associations were estimated with reasonable precision.

It should be noted that the four engagement dimensions exhibited relatively high intercorrelations in the present study (r = 0.446 to 0.745). Given these correlations, additional multicollinearity diagnostics were conducted in SPSS using a linear regression model, with French achievement as the outcome variable and the four engagement dimensions as simultaneous predictors. Variance Inflation Factors (VIFs) ranged from 1.658 (social engagement) to 3.032 (emotional engagement), all well below the conventional threshold of 5.0 (Hair et al., 2019). Condition indices ranged from 1.000 to 17.201; the two highest values (16.777 and 17.201) fell within the moderate range (10–30) (Belsley et al., 1980). While these linear regression-based checks provide additional reassurance that severe multicollinearity is absent, they do not fully capture the complexity of a latent-variable SEM and therefore do not eliminate the need for caution. The diagnostics show that the engagement dimensions shared a non-negligible amount of variance. The path coefficients reported in this study should therefore be interpreted cautiously as conditional effects; that is, they show the unique contribution of each dimension after accounting for the others, rather than independent direct effects.

### 5.3. Mediation Model Analysis

A confirmatory factor analysis (CFA) was conducted to evaluate the measurement model encompassing five latent variables: language attitudes, cognitive engagement, emotional engagement, behavioral engagement, and social engagement. The results indicated a good fit for the five-factor measurement model (χ^2^/df = 2.051, CFI = 0.942, TLI = 0.934, RMSEA = 0.056, SRMR = 0.057). The standardized factor loadings and Average Variance Extracted (AVE) for all latent variables were >0.5, and the Composite Reliability (CR) values were all >0.7, supporting the convergent validity and reliability of the measurement model. To provide stronger evidence of discriminant validity, the five-factor model was further compared with several more constrained alternative models in which the most highly correlated engagement dimensions were progressively merged (see Table 4). The five-factor model showed the best fit and the lowest AIC and BIC values among all models, with fit deteriorating progressively as dimensions were combined. These comparisons provide further support for the discriminant validity of the five-factor model, suggesting that the engagement dimensions remain empirically distinguishable despite their strong intercorrelations.

**Figure 2:**
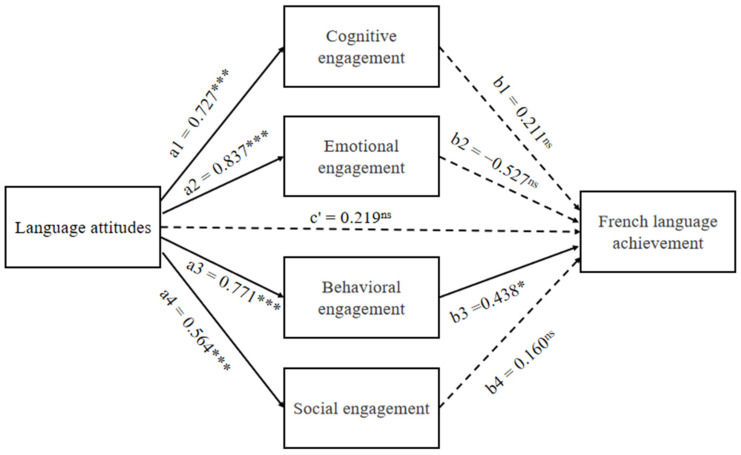
Structural equation modeling results. Note: All coefficients are standardized; the dotted line indicates a non-significant result. * *p* < 0.05, *** *p* < 0.001; ns = non-significant.

Based on the well-fitting measurement model, a parallel multiple mediation model was estimated. The results showed that the structural model also fitted the data well (χ^2^/df = 2.629, CFI = 0.934, TLI = 0.924, RMSEA = 0.070, SRMR = 0.056). As illustrated in Figure 2, language attitudes showed significant positive paths to cognitive engagement (β = 0.727, *p* < 0.001), emotional engagement (β = 0.837, *p* < 0.001), behavioral engagement (β = 0.771, *p* < 0.001), and social engagement (β = 0.564, *p* < 0.001). Among the four dimensions, only behavioral engagement showed a significant positive path to French achievement (β = 0.438, *p* = 0.024); the paths from cognitive, emotional, and social engagement to French achievement were not significant (*p* > 0.05). The direct path from language attitudes to French achievement was not significant (β = 0.219, *p* = 0.163).

Given the substantial intercorrelations among the engagement dimensions, the four engagement-to-achievement paths were estimated simultaneously; each coefficient therefore reflects the unique association of that dimension with French achievement after controlling for overlapping engagement variance. Accordingly, the non-significant paths for cognitive, emotional, and social engagement indicate that these dimensions did not show statistically significant unique associations with French achievement, rather than indicating an absence of association altogether. By contrast, behavioral engagement showed a significant positive path to French achievement after accounting for the other dimensions.

A bias-corrected bootstrap procedure (5000 resamples) was used to estimate the direct, total, and specific indirect effects (see Table 5). The total effect of language attitudes on French achievement was significant (β = 0.359, 95% CI [0.219, 0.488]). Among the four specific indirect paths, only the path through behavioral engagement was significant (β = 0.338, 95% CI [0.108, 0.672]); the paths through cognitive (β = 0.153, 95% CI [−0.018, 0.392]), emotional (β = −0.441, 95% CI [−1.117, 0.235]), and social engagement (β = 0.090, 95% CI [−0.021, 0.249]) were not significant, as their confidence intervals all included zero.

Notably, although emotional engagement was positively correlated with French achievement at the bivariate level (r = 0.296, 95% CI [0.194, 0.391]), its path to achievement in the multivariate model was negative (β = −0.527, ns), as was the corresponding indirect effect (β = −0.441, ns). Given the high intercorrelations among the engagement dimensions, this sign reversal plausibly reflects shared variance (a suppression pattern) among the mediators rather than a substantive negative relationship between emotional engagement and French achievement. Because this indirect effect was not statistically significant, it is not interpreted substantively here.

## 6. Discussion

### 6.1. Correlations Among Language Attitudes, Student Engagement, and French Achievement

The Pearson correlation analysis revealed significant positive correlations among all core variables. On the one hand, language attitudes were moderately to highly correlated with all four dimensions of student engagement. This suggests that learners with more positive attitudes toward the target language also tended to be more engaged cognitively, emotionally, behaviorally, and socially. A similar pattern has been reported in studies conducted in different cultural contexts (e.g., the Philippines) and among non-English major L2 learners (e.g., Candog et al., 2024; Yan, 2024). Compared to other dimensions, the correlations between language attitudes and both emotional and behavioral engagement were particularly prominent. This may reflect the characteristics of early adolescents’ cognitive developmental stages, wherein their reflective capacities and motivational regulation mechanisms are not yet fully mature (Caldwell-Harris & MacWhinney, 2023; Wesarg-Menzel et al., 2023). Consequently, the link between student attitudes and their engagement appears more straightforward. For example, a seventh-grade student might strongly believe that “French sounds friendly, comfortable, and attractive” (the affective dimension of language attitudes). This positive attitude is often closely accompanied by active behavioral manifestations. The participant “always looks forward to the next French class” and feels a “wish that the French classes could be longer when they are finished” (the emotional dimension of student engagement), while also more proactively choosing to “stick to French study schedule strictly” (the behavioral dimension of student engagement). Such associations likely occur without the complex internal reflection and regulation mechanisms typical of adults. In contrast, deep cognitive engagement requires higher-order metacognitive skills and strategic effort, which are not yet fully developed during early adolescence (Oga-Baldwin & Nakata, 2017; Park & Yun, 2017; Sung et al., 2016).

On the other hand, both language attitudes and student engagement were significantly and positively correlated with French academic achievement, which is consistent with previous findings (e.g., Eerdemutu et al., 2024; Li & Li, 2024; Zhong et al., 2023). Notably, cognitive and behavioral engagement showed stronger associations with French achievement, with their correlation coefficients being markedly higher than those of the other two dimensions. We postulate that this may be related to the comprehensive reading-writing tasks widely adopted in the Chinese foreign language teaching context. Given that reading and writing account for a substantial proportion of the academic achievement test, teachers typically emphasize continuation-writing training in daily instruction. This practice mode requires learners to engage in deep cognitive processing (e.g., comprehending and conceptualizing) and high-intensity behavioral output (e.g., writing practice). As C. Wang (2015) noted, by providing a source text, continuation-writing organically integrates language imitation with content creation, bridging reading input with writing output. Such tasks are thought to foster learners’ willingness to express themselves and to support meaningful language acquisition, as they facilitate the transition from receptive to productive skills.

### 6.2. The Mediating Role of the Four Dimensions of Student Engagement Between Language Attitudes and French Achievement

Another core finding of this study is that, within the parallel multiple mediation model, behavioral engagement emerged as the sole significant mediating variable linking language attitudes and French academic achievement. It highlights the role of “action” in language learning. As Li and Wei (2025) have observed, “The student behaves in accordance with his or her attitudinal evaluations of the language and language class” (p. 3326). That is, language attitudes are often expressed through learning behavior, which is in turn linked to scholastic achievement (Schaefer & McDermott, 1999). Specifically, although positive attitudes (e.g., liking French) may provide psychological energy, this energy appears to be reflected in test scores primarily when it is translated into concrete, observable learning behaviors (e.g., attendance, assignment completion, reading aloud practices), particularly in skill-output-oriented French tests. This pattern suggests that, under the current pedagogical evaluation system, explicit effort may be a salient factor associated with academic performance.

Furthermore, the direct path from language attitudes to French achievement was non-significant (β = 0.219, *p* > 0.05), indicating that the contribution of attitudes was largely indirect rather than direct. This result differs from the conclusions of Li and Wei (2025), a difference that may mainly stem from differences in model complexity and research focus. Through a parsimonious path model, Li and Wei (2025) aimed to establish the fundamental association between attitudes and achievement, thereby capturing the total effect. In contrast, the present study constructed a more granular and multidimensional “attitude-engagement-achievement” framework to examine the pathways through which language attitudes relate to French achievement. Within such a complex mediation model, once a more proximal correlate such as behavioral engagement is introduced, the direct contribution of language attitudes, as a more distal factor, may be attenuated and, in some cases, may no longer reach statistical significance. This does not negate the importance of attitudes; rather, it indicates that their role is better understood as “indirect” and “contextualized.”

Cognitive, emotional, and social engagement were not significantly associated with French achievement once all dimensions were entered simultaneously. Two factors may help account for this result. First, rather than indicating a lack of influence, this result likely reflects the competition for explanatory power among engagement dimensions, compounded by the implicit nature of these variables. In this study, behavioral engagement exhibited high, significant positive correlations with both cognitive and emotional engagement (r > 0.70; see Table 2), indicating a close coupling between students’ internal psychological states and explicit learning behaviors during the authentic L2 acquisition process. When these highly covarying variables simultaneously entered the model to compete for explanatory power over French achievement, behavioral engagement, as the most directly observable and readily quantifiable dimension, may have absorbed a large share of the variance, thereby reducing the residual explanatory variance available to cognitive and emotional engagement and attenuating their unique contributions to the outcome. This pattern is most evident for emotional engagement. Although emotional engagement was positively correlated with French achievement at the bivariate level (r = 0.296, *p* < 0.001), its path to achievement in the multivariate model was negative and non-significant (β = −0.527, ns), as was the corresponding indirect effect (β = −0.441, ns). Because emotional and behavioral engagement are highly intercorrelated (r = 0.731), the variance they share is largely captured by behavioral engagement, which plausibly drives the emotional-engagement coefficient negative. We interpret this sign reversal as a suppression pattern arising from shared variance among the mediators rather than as evidence that emotional engagement is substantively detrimental to French achievement; because the effect was not statistically significant, we do not interpret it substantively. Pedagogically, this may reflect the disparity between implicit psychology and explicit skills. Cognitive and emotional engagement largely pertain to implicit psychological states, whereas Chinese academic achievement tests (such as French examinations) primarily assess hard skills like vocabulary, grammar, and reading-writing. The development of these skills, particularly as reflected in formal French achievement, may be more closely associated with repetitive practice and behavioral engagement than with psychological endorsement or emotional pleasure alone. As Yang (2011) has asserted, “language acquisition requires repetition and stimulation; fleeting information necessitates repeated reinforcement before it can potentially be transformed into long-term memory storage” (p. 32). In other words, high cognition (e.g., strategy use) and high emotion (e.g., joyful experiences) may be more likely to relate to academic outcomes when accompanied by behavioral manifestation, positioning behavioral engagement as a statistical proxy for the more implicit dimensions in this model. We acknowledge, however, that this interpretation remains inferential. The multicollinearity diagnostics (Section 5.2) provide additional reassurance in a technical sense (VIFs = 1.658–3.032; max condition index = 17.201), yet the high inter-dimensional correlations (r = 0.73–0.90) suggest that the individual path estimates may still be sensitive to shared variance among the mediators, and therefore do not eliminate the need for caution. Future studies could further examine these relationships using alternative model specifications, such as higher-order engagement models, sequential mediation models, or bifactor models, to better distinguish the common and dimension-specific components of engagement.

Second, features of the present learners’ linguistic proficiency and instructional context may further help explain why social engagement, in particular, was not significantly associated with French achievement. Much engagement research to date has been conducted with university-level EFL learners (e.g., Alrashidi & Alshammari, 2024; Tong & Singh, 2025), who typically possess sufficient communicative competence in the target language; by contrast, the junior secondary FFL learners in the present study are mostly at the beginner stage of language acquisition. Their limited vocabulary and grammatical repertoire make it more challenging for them to genuinely use French for deep group discussions or immersive teacher-student interactions in the classroom. Consequently, social engagement may not be reflected in written test scores as directly as it might in more proficient or communicatively oriented learning contexts. Furthermore, regarding junior high school French instruction, most schools are driven by high-stakes entrance examination preparation (e.g., the High School Entrance Examination) (Xiao, 2017). Against this backdrop, pedagogical models tend to be traditional, relying heavily on one-way teacher instruction or individualized study, with a relative scarcity of collaborative learning task designs. Under such conditions, social-level learning participation (e.g., eagerness to ask questions or interact with peers) is difficult to fully stimulate, thereby further attenuating its observed relationship with individual test scores.

## 7. Conclusions

Focusing specifically on Chinese junior high school FFL learners, this study found that language attitudes were not directly linked to L2 achievement; rather, their association with achievement operated primarily through behavioral engagement. In other words, at this developmental stage, early adolescents tend to perform better academically when their positive attitudes are expressed through concrete learning actions.

The findings offer several theoretical implications. First, this study supported the mediation pathway of “language attitudes → behavioral engagement → FL achievement.” This extends the proposed mechanism of Li and Li (2024) regarding the association between language attitudes and FL academic achievement by showing that, beyond L2 emotions, additional factors are involved in this relationship. Second, the research refines the construct of student engagement by providing evidence that different dimensions of engagement do not relate equally to L2 academic achievement, thereby establishing a more granular analytical framework for the field. Furthermore, the findings highlight the value of adding language attitudes—a classic sociolinguistic concept—into the inventory of individual difference factors in SLA (Gardner et al., 1997). This is consistent with CVT’s perspective that language attitudes, serving as value appraisals in the context of language learning, together with control appraisals, constitute distal factors bearing on academic achievement. More importantly, by providing evidence for the role of language attitudes among early adolescent FFL learners in China, this study broadens the research scope beyond the traditional focus on English users, enriching valuable cross-linguistic and cross-age empirical evidence in the field of L2 attitudes and motivation.

Pedagogically, the findings offer actionable insights for multilingual education at the foundational level. Teachers can intervene at two complementary levels. On the one hand, given the malleability of language attitudes (Smith, 1971), FFL teachers can cultivate young beginners’ positive language attitudes through multicultural teaching, scenario-based task design, and personalized feedback. On the other hand, because junior high school students’ complex self-regulation and metacognitive capacities are not yet fully developed, merely stimulating their interest in French appears far from sufficient. Pedagogical practices should also place strong emphasis on stimulating and maintaining “behavioral engagement.” Teachers can employ actionable and quantifiable explicit strategy instruction to guide learners in translating their psychological advantages into sustained explicit learning behaviors. Examples include integrating language skill training into comprehensive communicative tasks (Gao, 2017) and achieving the effective integration of reading and writing (J. Zhang, 2022).

Several limitations warrant acknowledgment. First, the cross-sectional design cannot definitively establish the causal direction among variables. Future studies could employ longitudinal or experimental intervention designs to capture the dynamic changes in engagement states. Furthermore, the single-site-per-city sampling risks unexamined clustering effects; follow-up work incorporating multilevel modeling would help to address this issue. In addition, although model parsimony is prioritized, key covariates like gender and grade level are not included, which may limit the developmental sensitivity of the findings. Finally, given the relatively small sample, subsequent research should be conducted on a larger scale in order to further investigate whether learning motivation, cognition, and learning strategies play mediating roles between language attitudes and FL achievement. Such a study would illustrate the factors associated with LOTE achievement more comprehensively and deeply.

## Figures and Tables

**Table 1:** Descriptive statistics and normality test results. Note. French achievement is reported as raw scores for interpretability. All subsequent analyses utilized grade-standardized z-scores.

Variables	M	SD	Min	Max	Skewness	SE	Kurtosis	SE
Language attitudes	5.42	1.34	1	7	−1.06	0.13	0.76	0.27
Overall student engagement	3.63	0.86	1	5	−0.74	0.13	0.56	0.27
Cognitive engagement	3.70	0.99	1	5	−0.74	0.13	0.21	0.27
Emotional engagement	3.66	0.98	1	5	−0.59	0.13	−0.08	0.27
Behavioral engagement	3.44	1.01	1	5	−0.51	0.13	−0.13	0.27
Social engagement	3.85	1.05	1	5	−0.91	0.13	0.30	0.27
French achievement	70.00	17.02	8	100	−0.95	0.13	0.77	0.27

Note: M = Mean; SD = Standard Deviation; SE = Standard Error.

**Table 2:** Correlation Matrix Among Language Attitudes, Student Engagement (and its Four Dimensions), and French Achievement.

Variables	1	2	3	4	5	6	7
1. Language attitudes	-	-	-	-	-	-	-
2. Overall student engagement	0.769 ***	-	-	-	-	-	-
3. Cognitive engagement	0.675 ***	0.899 ***	-	-	-	-	-
4. Emotional engagement	0.717 ***	0.886 ***	0.745 ***	-	-	-	-
5. Behavioral engagement	0.721 ***	0.890 ***	0.739 ***	0.731 ***	-	-	-
6. Social engagement	0.487 ***	0.726 ***	0.560 ***	0.602 ***	0.446 ***	-	-
7. French achievement	0.328 ***	0.382 ***	0.366 ***	0.296 ***	0.365 ***	0.248 ***	-

Note: *** *p* < 0.001.

**Table 3:** Main Correlations and 95% Confidence Intervals.

Variable 1	Variable 2	r	95% CI	*p*
Language attitudes	Overall student engagement	0.769	[0.721, 0.809]	<0.001
Language attitudes	Cognitive engagement	0.675	[0.613, 0.730]	<0.001
Language attitudes	Emotional engagement	0.717	[0.660, 0.765]	<0.001
Language attitudes	Behavioral engagement	0.721	[0.665, 0.769]	<0.001
Language attitudes	Social engagement	0.487	[0.401, 0.565]	<0.001
Language attitudes	French achievement	0.328	[0.228, 0.422]	<0.001
Overall student engagement	French achievement	0.382	[0.285, 0.471]	<0.001
Cognitive engagement	French achievement	0.366	[0.268, 0.457]	<0.001
Emotional engagement	French achievement	0.296	[0.194, 0.391]	<0.001
Behavioral engagement	French achievement	0.364	[0.266, 0.455]	<0.001
Social engagement	French achievement	0.248	[0.144, 0.346]	<0.001

Note: Values are Pearson correlation coefficients. Confidence intervals were calculated using Fisher’s r-to-z transformation. CI = confidence interval.

**Table 4:** Comparison of Alternative CFA Measurement Models.

Model	Chi-Square (df)	CFI	TLI	RMSEA	SRMR	AIC	BIC
Five-factor	537.4 (262)	0.942	0.934	0.056	0.057	22,464.9	22,800.6
E + B combined	607.5 (266)	0.928	0.919	0.062	0.062	22,554.7	22,875.1
C + E + B combined	803.7 (269)	0.888	0.875	0.077	0.064	22,817.0	23,125.9
S + C + E + B combined	956.4 (271)	0.856	0.841	0.087	0.072	23,022.0	23,323.4
Single-factor	1592.7 (275)	0.724	0.699	0.120	0.076	23,927.1	24,213.2

Note: Models were estimated using MLR. The five-factor model specified language attitudes and the four engagement dimensions as distinct latent constructs. In the alternative models, language attitudes were retained as a separate factor while the engagement dimensions were progressively merged. The single-factor model loaded all items onto one general factor. C = cognitive engagement; E = emotional engagement; B = behavioral engagement; S = social engagement. Lower AIC and BIC values indicate better fit.

**Table 5:** Standardized Mediation Effects and Bootstrap Confidence Intervals.

Effect	Path	Std. Estimate	SE	*p*	95% Bootstrap CI
Direct effect	Language attitudes -> French achievement	0.219	0.157	0.163	[−0.053, 0.505]
Total effect	Language attitudes -> French achievement	0.359	0.068	<0.001	[0.219, 0.488]
Total indirect	Language attitudes -> engagement dimensions -> French achievement	0.140	0.138	0.307	[−0.116, 0.368]
Specific indirect	Language attitudes -> Cognitive engagement -> French achievement	0.153	0.114	0.178	[−0.018, 0.392]
Specific indirect	Language attitudes -> Emotional engagement -> French achievement	−0.441	0.342	0.197	[−1.117, 0.235]
Specific indirect	Language attitudes -> Behavioral engagement -> French achievement	0.338	0.157	0.031	[0.108, 0.672]
Specific indirect	Language attitudes -> Social engagement -> French achievement	0.090	0.073	0.214	[−0.021, 0.249]

Note: Standardized estimates are reported. Confidence intervals are 95% bias-corrected bootstrap intervals based on 5000 bootstrap resamples. SE = standard error; CI = confidence interval.

**Figure A1:**
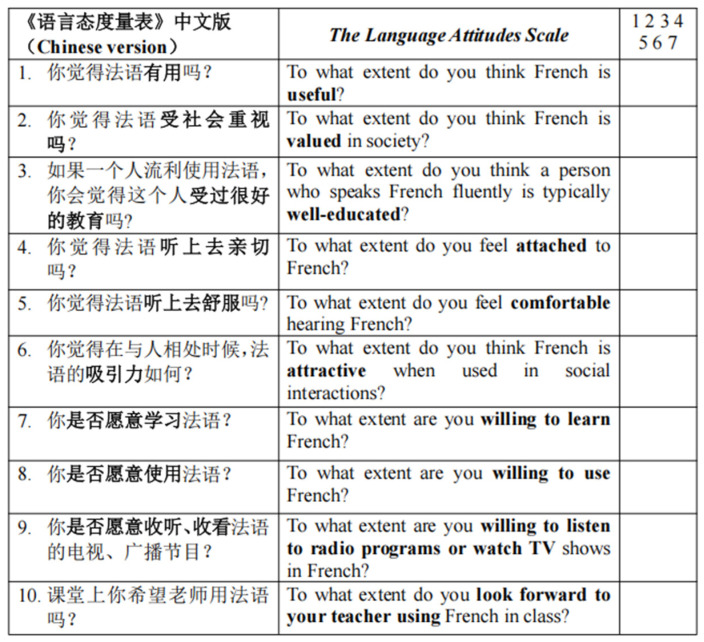
Language Attitudes Scale.

**Figure A2:**
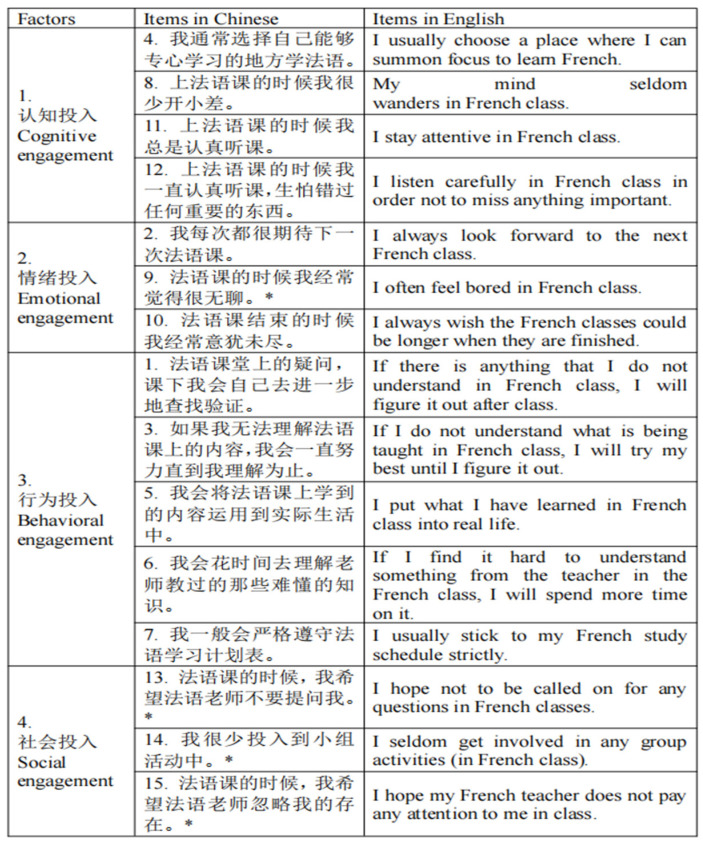
Student Engagement Scale. Note. * Items require reverse coding.

## Data Availability

The raw data supporting the conclusions of this article will be made available by the authors on request.

## Abbreviations

The following abbreviations are used in this manuscript:

FFL	French-as-a-foreign-language
EFL	English-as-a-foreign-language
FL	Foreign language
LOTE	Languages Other Than English
SLA	Second language acquisition
CVT	Control-Value Theory
